# The role of chiropractic care in older adults

**DOI:** 10.1186/2045-709X-20-3

**Published:** 2012-02-21

**Authors:** Paul E Dougherty, Cheryl Hawk, Debra K Weiner, Brian Gleberzon, Kari Andrew, Lisa Killinger

**Affiliations:** 1 Research Department, New York Chiropractic College, 2360 State Route 89, Seneca Falls, NY 13148, USA; 2 School of Medicine and Dentistry, University of Rochester, Rochester, NY 14620, USA; 3 Logan College of Chiropractic, Chesterfield, MO 63017, USA; 4 Anesthesiology & Psychiatry, VA Pittsburgh, Pittsburgh 15261, USA; 5 U. of Pittsburgh, 3550 Terrace Street Pittsburgh, PA 1526, USA; 6 Canadian Memorial Chiropractic College, 6100 Leslie Street, Toronto, ON M2H 3J1, Canada; 7 Palmer College of Chiropractic, 1000 Brady St, Davenport, IA 52803, USA

## Abstract

There are a rising number of older adults; in the US alone nearly 20% of the population will be 65 or older by 2030. Chiropractic is one of the most frequently utilized types of complementary and alternative care by older adults, used by an estimated 5% of older adults in the U.S. annually. Chiropractic care involves many different types of interventions, including preventive strategies. This commentary by experts in the field of geriatrics, discusses the evidence for the use of spinal manipulative therapy, acupuncture, nutritional counseling and fall prevention strategies as delivered by doctors of chiropractic. Given the utilization of chiropractic services by the older adult, it is imperative that providers be familiar with the evidence for and the prudent use of different management strategies for older adults.

## Introduction

By 2030, nearly one in five U.S. residents is expected to be age 65 or older [1]. An estimated 14% of patients treated by doctors of chiropractic (DCs) are 65 and older [2]. The most common reason for an older adult to see a DC is musculoskeletal pain, most often lower back pain [3]. Although the most common reason for older adults seeking chiropractic care is for musculoskeletal symptoms, DCs may also provide a diverse range of services to these patients [4]. Given this fact, for the purpose of this manuscript chiropractic care will be defined as; "the provision by a doctor of chiropractic of services related to patient assessment, maintenance of health, prevention of illness, and treatment of illness or injury". The focus of this manuscript is to describe the evidence for achievement of some of these goals in the older adult population. The purpose of this manuscript is to present an overview of information to the practicing chiropractor on utilization of specific management tools. This is not meant to be a systematic review of the literature or an evidence based guideline. The authors each have personal experience in evaluating and treating older adults as well as established expertise in research and publication in these areas. The authors recognize that there is a need for further research in the area of management of the older adult by DC's and discuss in the conclusion future research considerations.

Although chiropractic encompasses many different treatment modalities, the authors have chosen to focus on five specific interventions that are commonly utilized by DCs: spinal manipulative therapy (SMT), acupuncture, physical activity/exercise, nutritional counseling and fall prevention [5,6]. These have been chosen not only because they are commonly utilized treatments, but also because they align with certain goals of Healthy People 2020: 1) to reduce the proportion of older adults with functional limitations and 2) to increase the proportion of older adults with reduced physical or cognitive function engaging in physical activities; 3) to reduce the number of falls among older adults [7,8].

Many older adults utilize chiropractic services throughout the US and Canada. A recent longitudinal study reported 14.6% utilization over a 15 year period (1993-2007) with an annual prevalence rate of between 4.1%-5.4% [2]. The majority of older adults seek chiropractic care for back and/or neck pain, and treatment approaches for these conditions vary widely. Most DCs utilize some form of SMT. More than 90% provide nutritional advice and recommend nutritional supplements. Most also recommend therapeutic exercises and advise patients to engage in physical activity [5,6]. There is also a growing trend in the utilization of acupuncture in older adults [9]. Although few studies have specifically evaluated the role of chiropractic care in older adults, it is imperative that practicing DCs familiarize themselves with the unique nuances of dealing with older adults and understand the evidence for treatment approaches. The authors provide a brief overview of the current evidence for each of the interventions listed above as well as their commonly reported indications and contraindications.

### Role of spinal manipulative therapy in older adults

Spinal pain is a significant musculoskeletal problem among older patients [10]. A recent report states the prevalence of disabling and non-disabling back pain in community-dwelling adults is 6% and 23%, respectively [11]. There are data that suggest that SMT may play an important role in the management of patients with spinal and peripheral joint pain and associated dysfunction [12]. From the perspective of the public, chiropractic care is most closely associated with SMT, which is traditionally high-velocity, low-amplitude (HVLA) maneuvers applied manually to spinal and peripheral joints [13]. These maneuvers move the joints from the end of their active and passive ranges of motion into the paraphysiological joint space, but not beyond their limit of anatomic integrity to deliver a therapeutic stimulus to the joint complex [14].

It is important to recognize that SMT used by DCs in practice may incorporate multiple techniques. These techniques may include varying levels of biomechanical force, ranging from high-velocity, low-amplitude to low-velocity, low-amplitude. SMT may also include instrument-assisted manipulation, use of specialized tables, use of padded wedges and many low-force techniques [5,6]. While it is assumed that alteration of techniques may play an important role in older adults where variation in technique and application of force is thought to aid in prevention of injury associated with a SMT, there is a need for further research to determine the most appropriate approach in this population. There are two trials that have compared outcomes in older adults comparing a higher force technique versus a lower force technique; both of these trials demonstrated comparable results with both techniques [15,16].

There is a limited but suggestive body of knowledge that supports the effectiveness of SMT for many conditions that affect older patients [17]. This body of knowledge includes expert opinion, case reports, case series, observational studies and a few randomized controlled trials [17]. The majority of studies evaluated the role of SMT for musculoskeletal pain syndromes including lower back pain with and without stenosis [15,18] neck pain with and without stenosis [19,20], knee pain [21,22] and thoracic function [23,24]. In addition there has been investigation into the role of SMT in patients with dizziness [25-28].

There are a number of case reports and case series that describe the successful management of older patients with spinal or peripheral joint pain using low-velocity, low-amplitude mobilizations and high-velocity, low-amplitude manipulations as well as instrument-assisted manipulation, padded wedges and other low force techniques [29]. Observational studies and randomized controlled trials have reported improvement of spinal pain (acute, sub-acute and chronic) among seniors using not only SMT but also low force techniques such as BioEnergetic Synchronization Technique [16] and Cox Flexion-Distraction technique [15]. The 2010 UK Report of Manual therapies reported that "SMT is effective in adults for: acute, subacute, and chronic low back pain; migraine and cervicogenic headache; cervicogenic dizziness; manipulation/mobilization is effective for several extremity joint conditions; and thoracic manipulation/mobilization is effective for acute/subacute neck pain [30]." However, the authors did not do a sub-analysis of older adults.

In addition to spinal pain, there is a limited body of evidence reporting on the role of SMT to address symptoms associated with other co-morbid clinical conditions [31], including: COPD [23,24], constipation [32], depression (associated with back pain) [33], Parkinson's disease [34,35], multiple sclerosis [36], pneumonia [37], spinal stenosis [18], urinary incontinence [38], and osteoarthritic pain and dysfunction, especially of the knee [21]. This body of evidence is limited in that is it chiefly comprised of descriptive studies--case reports and case series. There are no high quality trials reporting on these conditions. There is a need to further investigate the role of SMT in the older adult for these non-spine related conditions.

#### Safety

There are limited data on the safety of SMT in the older adult population. Dougherty et al. have reported on the safety of SMT in the older adult population, specifically in osteoporosis, anticoagulation therapy and spinal stenosis. These data are from two randomized controlled trials and also from retrospective data from a chiropractic clinic in a long term care facility. The retrospective file review reports on institutionalized older adults who underwent SMT. These patients had significant co-morbidities such as osteoporosis, post-stroke status, Parkinson's disease, amyotrophic lateral sclerosis and other chronic conditions [34]. The data from the randomized controlled trials showed adverse event data lower than previously reported in the literature, with only 35% of the patients experiencing any type of AE and none experiencing any serious adverse events associated with SMT [39]. It is noted that the AE's reported in this trial utilized a standard definition of AE's utilized typically in drug trials. However, these were reported utilizing different terminology than the retrospective review of older adults treated in a long-term care facility, which used recently reported manual therapy specific terminology for reporting AE's [40]. This variation in criteria for reporting adverse events highlights the need to develop a universally accepted format of reporting adverse events in studies evaluating spinal manipulation that can be used in multiple research settings.

It is felt that there may even be a lower incidence of AE's associated with SMT in older adults due to the practice by most DCs of modifying their use of SMT, often substituting lower-force for higher-force procedures to minimize the net force applied to a joint [41]. In addition to the topics discussed above, there are other non-manipulative manual procedures available to DCs [29,42]. Table 1 describes the results of a consensus project on best practices in the geriatric population concerning SMT treatment considerations in the older adult [4].

**Table 1:** Treatment considerations for older adults [4]

Patient characteristic	Treatment modification
Small body size; increased frailty	Decrease biomechanical force
Severe osteoporosis and/or other bone diseases such as infection or cancer.	High-force manipulation is contraindicated
Anticoagulation or corticosteroid medication	Soft tissue or other manual procedures which compress tissues are either contraindicated or should be used with caution and decreased biomechanical force.
Fear or anxiety related to manipulation, or preference for type of procedure	Adapt manipulation and soft tissue procedures to optimize patient comfort

### Acupuncture in older adults

For the purpose of this manuscript, "acupuncture" is defined as any of a variety of interventions that are administered using acupuncture needles. In the setting of low back pain management, this includes but is not limited to traditional Chinese acupuncture, percutaneous electrical nerve stimulation (PENS; a.k.a. percutaneous neuromodulation therapy), auricular acupuncture, trigger point deactivation, and deep intramuscular electrical stimulation. It is of note that there have been no trials conducted in which chiropractors have delivered the acupuncture to older adults. There is a need to further evaluate the role of chiropractors delivering acupuncture in a clinical setting, particularly in older adults who may not be able to tolerate more traditional manual therapy techniques. There is also a need to standardize the role of training of chiropractors to perform acupuncture in older adults. The authors have chosen to only discuss those trials that have specifically focused on older adults with LBP, since that is the most common presentation of older adults to chiropractors.

While older adults have been included in some trials evaluating the efficacy of acupuncture for the treatment of low back pain (LBP), only two have focused exclusively on older adults [43,44]. These have evaluated a more contemporary acupuncture technique, lumbar PENS. The first of these studies was a randomized controlled pilot trial involving 34 community dwelling older adults with chronic low back pain (CLBP) who received either lumbar PENS or a control procedure (acupuncture needles without electrical stimulation) twice a week for 6 weeks and participants were followed for 3 months [43]. Both groups also received physical therapy. Those randomized to lumbar PENS experienced significant pain reduction and functional improvement that was maintained at 3 months, but the group that received the control procedure experienced no improvement [43].

The second study was a randomized controlled trial of 200 older adults with CLBP who received one of four interventions twice a week for 6 weeks: 1) lumbar PENS and a general conditioning and aerobic exercise (GCAE) program, 2) lumbar PENS alone, 3) limited lumbar PENS (only 2 of 12 needles were stimulated for 5 minutes as compared with full PENS during which all needles are stimulated for 30 minutes), or 4) limited lumbar PENS and GCAE. Participants were followed for 6 months and all four groups experienced significant improvement that was maintained during the follow-up period [44]. Thus while electrical stimulation appears to be essential for therapeutic response, the minimal therapeutic dose is unknown. In addition to treatment for lower back pain, other studies have evaluated the role of acupuncture for other conditions. A recent review of the use of acupuncture for chronic musculoskeletal pain reported that there is insufficient experimental evidence to recommend the use of traditional Chinese acupuncture over other modalities for older adults with persistent musculoskeletal pain. The authors then state however that there are promising preliminary data to support the use of percutaneous electrical nerve stimulation for persistent low back pain [45].

The other acupuncture procedures noted above have not been studied exclusively in older adults, although they are not uncommonly used by practitioners trained in acupuncture. With Traditional Chinese Acupuncture, needles are strategically placed in acupuncture points to move the flow of energy or Qi (pronounced "chi"). Acupuncture points may be distant from the site of pathology. Needles may be stimulated mechanically (i.e., the acupuncturist intermittently turns the needles), with heat (e.g., lamp, moxibustion), light (e.g., laser), or electricity. Percutaneous electrical nerve stimulation uses electricity exclusively, and needles are placed neuroanatomically along dermatomes, myotomes and scleratomes. Duration of treatment with TCA and PENS is typically 20-40 minutes. Auricular acupuncture guides the placement of acupuncture needles according the homunculus on the ear's surface. A variety of needle types are available, some of which can be left in place for several days. Trigger point deactivation ("dry needling") and deep intramuscular electrical stimulation are targeted to localized areas of myofascial pain. There is a need for further research in evaluating the therapeutic role of these specific techniques in older adults.

A summary of the indications, relative contraindications and potential adverse effects associated with these procedures is provided in Table 2.

**Table 2:** Acupuncture and related modalities for the treatment of CLBP

Modality	Indications	Contraindications (relative)	Potential Adverse Effects
Traditional Chinese Acupuncture (TCA)	Insufficient clinical trials evidence to recommend for older adults with CLBP	• Bleeding diathesis • Pacemaker (if leads cross chest when using electrical stimulation) • Severely immune-compromised state	• Bleeding, bruising • Infection (very rare) • Transient pain flare • Transient fatigue • Vasovagal response
Percutaneous electrical nerve stimulation (PENS)	CLBP in older adults; minimum effective dose of electrical stimulation unknown	As with TCA	As with TCA
Auricular acupuncture	Insufficient clinical trials evidence to recommend for older adults with CLBP; theoretically useful for augmenting corporeal treatments.	• Bleeding diathesis • Cartilage disease (e.g., relapsing polychondritis)	As with TCA
Trigger point deactivation	Myofascial pain; local twitch response is essential therapeutic element (ref). May be combined with other acupuncture modalities, e.g., following PENS for recalcitrant localized pain.	As with TCA	As with TCA; as compared with TCA and PENS, trigger point deactivation may be painful.
Deep intramuscular electrical stimulation	Localized myofascial pathology (e.g., piriformis, erector spinae); may be performed in addition to TCA or PENS for recalcitrant localized myofascial pain.	As with TCA	As with TCA

### Nutritional counseling in older adults

Current research indicates that a large percentage of older adults do not receive adequate amounts of micronutrients in their daily diet [46,47]. Studies report that prevention strategies in the form of improvement in diet and health promotion counseling can lead to improved quality of life, significant reductions in disability, and reduction in health care costs [48,49].

The main goal for nutritional counseling should be to improve food choices, particularly with respect to increasing the intake of fruits and vegetables [48]. If consumption of adequate energy and micronutrients cannot be managed with dietary modification the use of dietary supplements may be considered. However, given the lack of rigorous clinical trials evaluating the effectiveness of dietary supplements it is difficult to make strong recommendations for or against nutritional supplements for meeting the nutritional needs of older adults.

Although multivitamin-mineral (MVM) supplements are used commonly by older adults, there is limited evidence of their impact on health outcomes [50]. Vitamin D and calcium supplements appear to have the most beneficial effects [51,52]. They have been found to be a critical adjunct to any pharmacologic regimen in the treatment of osteoporosis [52] and in the prevention of hip fractures and other non-vertebral fractures [51]. In addition, a recent systematic review on Vitamin D found that "Vitamin D treatment effectively reduces the risk of falls in older adults [53]." Recommended daily intake for calcium and vitamin D are 1,200 mg and 1,000 IU respectively [51]. All other commonly consumed supplements cannot be recommended at this time either due to inadequate evidence or due to evidence of significant side effects.

Even though a supplement may be considered "benign," providers must be aware of potential drug or disease interactions [51]. Data from the National Social Life, Health and Aging Project (NSHAP), a recent population-based survey of community-dwelling older adults in the United States, evaluated the potential impact of medication use on clinical outcomes that may result from drug interactions, including interactions between prescription and nonprescription therapies. Among prescription medication users, concurrent use of over-the-counter medications was 46% and concurrent use of dietary supplements was 52%.

A recent survey on dietary supplement use in the U.S. found that about one-half of the U.S. population and 70% of adults ≥ 71 years of age use dietary supplements; one-third use multivitamin-multimineral dietary supplements [54]. Overall, 68% of older adults using prescription medications are concurrently using over-the-counter medications, dietary supplements, or both. It was found that 1 in 25 older adults (approximately 2.2 million) were at risk for a major potential drug-drug interaction, and that more than half of these involved non-prescription therapies (including supplement use) [55]. Physicians are frequently unaware of their patients' nonprescription medication and/or supplement use because they do not ask patients; patients do not report such use, or both. The economic and health consequences of these potential interactions are considerable [55]. It is therefore imperative to for both the consumer and the DC to understand the benefits and risks for utilization of dietary supplements, identify potential interactions with other medications, and avoid large combinations to decreased overconsumption [52]. Adverse events associated with supplement use alone, apart from interactions, may include excess levels of iron and zinc. In addition, iron overload has been associated with coronary artery disease. Zinc overload has been associated with reduced copper status, impaired immune response, and lowered plasma high-density lipoprotein cholesterol levels [46].

These findings suggest that encouraging positive attitudes about a healthful diet in combination with nutrition guidance and appropriate supplement information may result in promoting advantageous use of supplements by this at-risk population.

### Physical activity and exercise in older adults

The majority of DCs report that they utilize therapeutic exercise in their practices.^5 ^Physical activity and exercise are often recommended by DCs to their older adult patients. A recent population survey demonstrated that exercise recommendation was underutilized by providers of multiple disciplines [56]. Position statements for osteoarthritis recommend exercise as a key component of disease management [57]. Painful limitation of function and muscle loss (i.e., sarcopenia) are two common reasons for the recommendation of exercise in this population. The loss of muscle strength has been identified as a physiologically limiting factor to living independently among older persons [58]. Recent reviews have shown the positive effects of aerobic exercise and strength training on strength, balance, and physical functioning [59]. These reviews demonstrate a modest beneficial effect of resistive training on strength outcomes and strong evidence for the improving function, particularly gait speed and chair stands.

There are a few studies that have specifically evaluated long term improvements in disability in patients who undergo resistance training [60]. There are also strong data supporting the role of resistance exercise in improving pain associated with knee osteoarthritis [61]. A recent study reports that community dwelling older adults who were adherent to an adaptive physical activity program had lower levels of pain related disability [62]. Back pain is a common problem among the older adult population and the most common condition for which older adults seek care with a DC. DCs often combine SMT and exercise therapy. Recent studies have demonstrated the benefits of combining exercise and spinal manipulation for chronic lower back pain; however none of these studies have been specifically carried out in older adults [63].

A recent comparative effectiveness review found structured exercise and SMT appear to offer equivalent benefits in the management of pain and function for patients with nonspecific chronic LBP [64]. The recommendation from this review was, "if no clinical benefit is appreciated after using one of these approaches for 8 weeks, then the treatment plan should be reevaluated and consideration should be given to modifying the treatment approach or using alternate forms of care [64]." In addition to the use of exercise for the treatment of back pain, a recently systematic review found that there is moderate evidence on the use of exercise for fall prevention [65]. Another systematic review found that interventions with balance exercises reduced falls or fall-related fractures and improved balance in the majority of the studies reviewed. Muscle strengthening exercises were also found to be effective in improving lower extremity strength and back extensor strength; however, not all RCT's reviewed reported positive effects. Bone strength was improved by weight-bearing aerobic exercise with or without muscle strengthening exercise when the duration of the intervention was at least a year [66].

Given the evidence for the use of exercise recommendation, it is important that the clinician be aware of any risks associated with exercise therapy in older adults. A recent review article found that among 121 trials identified, 53 trials provided no comments about adverse events, 25 trials reported no adverse events occurred, and 43 trials reported some types of adverse events. Most adverse events reported were musculoskeletal problems such as muscle strain or joint pain. Adverse events were reported more often in trials that recruited participants with certain health conditions, functional limitations, or sedentary lifestyle [67]. The most important considerations in providing an exercise program for older adults is to assess their general health status and to assure that the program is tailored to their needs, whether it is strength, endurance, balance or improved motion. The older adult should be educated in warning signs such as chest pain or shortness of breath.

The most important principle in developing an exercise program for the older adult is to work with him/her in order to agree on a program to which they will adhere to. Patient centered exercise prescription involves shared goals between the patient and the provider [68]. The practitioner may also want to perform simple functional tests periodically, such as timed up and go or single leg stance [69], to demonstrate to the patient that they are making progress, which may otherwise be difficult for the patient to perceive. Finally, patients should be encouraged to participate in activities they enjoy; in fact one study found that leisure time activity was more effective than structured exercise in patients with lower back pain [70].

### Fall prevention in older adults

Falls can result in decreased quality of life, disability, and/or death in older adults. Approximately one-third of those aged 65 and older fall each year, and falls are the leading cause of unintentional injuries and unintentional injury deaths in this population [71]. Related direct medical costs are at least $19 billion, expected to reach $44 billion by 2020 [65]. Of those fallers whose injuries require hospitalization, 40-50% subsequently lose their independence and enter a nursing home [72].

Fall prevention in older adults is a national priority, and is the subject of two *Healthy People 2020 *objectives [8]. Fall prevention interventions are based on the identification and reduction of modifiable risk factors. The most important modifiable risk factors for falls in older adults are impaired mental status; psychotropic medications; polypharmacy; environmental hazards; poor vision; lower extremity weakness and/or dysfunction; and impairments in balance, gait and activities of daily living [73].

A 2010 systematic review funded by the Agency for Healthcare Research and Quality examined interventions designed to reduce falls in older adults [65]. Physical therapy, Vitamin D supplementation and, as stated above in the section on physical activity, exercise interventions to improve gait, balance, and/or function were found to reduce fall risk [65]. Although chiropractic care was not included in the review, these interventions are within the scope of chiropractic practice.

A consensus statement on "best practices" for chiropractic care for older adults specifically recommends that DCs collect falls history information, and states that they provide treatment and exercises for musculoskeletal conditions, which can be extrapolated to be appropriate for reducing fall risk [4]. Furthermore, recent reviews written by DCs emphasize the importance of incorporating fall prevention into chiropractic practice [74,75].

Pain-related musculoskeletal disability may influence balance, gait and the ability to accomplish daily activities, and therefore may contribute to fall risk. There is considerable evidence for positive effects of SMT for spine-related pain [30]. Because chronic musculoskeletal pain, such as that of osteoarthritis, is one factor affecting gait and balance in older people, chiropractic care may have an impact on fall prevention by treating joint pain and stiffness. Also, the literature suggests a possible positive effect of SMT on certain types of vertigo. There is limited, although promising, evidence that manual therapies may be beneficial for cervicogenic vertigo [27,28,30,31,76,77].

## Discussion

DCs can play an important role in the management of health conditions in the older adult. Chiropractic care has often been associated only with the management of musculoskeletal disorders by the application of SMT. However, as we have discussed in this article, DCs often utilize multiple treatment modalities that address the patient as a whole, not only his or her musculoskeletal symptoms. It is important that the clinician evaluate the patient for any associated risks to the chosen intervention. There is a need for the practicing clinician to evaluate the most effective clinical strategy for managing the challenging older adult patient. The management of older adults is complicated by many factors such as co-morbidities that may limit the type of treatment intervention employed. These co-morbidities, as highlighted in this manuscript, include concerns of osseous weakness, interaction of nutritional supplements with current prescription medication as well as concerns about fall risk. The busy practitioner must plan to spend extra time with the older adults patient to appropriately manage this population. This is a challenge given the current healthcare system. A recent survey discussed the barriers to such things as fall prevention discussions with older adults [78]. Not only are there barriers to discussions about fall prevention, but there are concerns about the amount of time that should be taken to appropriately educate the older adult concerning exercise recommendations [79]. There is a strong case to be made for the role of multidisciplinary care in the older adult with musculoskeletal pathology, however to achieve this the practicing chiropractor must develop relationships with those outside the chiropractic profession. This integrated care model has been described and has the potential to improve the overall role of chiropractic in the healthcare system [80].

There is concern about risk in the chiropractic management of the older adult and as mentioned in the body of the manuscript there is a need for systematic evaluation of the risk associated with any management strategy. In order to facilitate this evaluation of risk there is a need to develop a common terminology for the reporting of untoward events associated with any management strategy. While there have been efforts toward this, unfortunately the proposed terminology proposed by Carnes et al. [40] are not practical in some research settings such as the Veteran's Health Administration.

Once risks have been ruled out, the DC, while being sensitive to patient preferences, can choose from a wide variety of treatment approaches within the chiropractic scope of practice. It is noteworthy that there remains a controversy concerning the role of the chiropractor in delivering acupuncture. In the US the regulations concerning the use of acupuncture vary by state and therefore it is not able to be utilized in all clinical settings. There is also a need for research further defining the role of acupuncture in the management of older adults.

As the number of older adults presenting to chiropractic practices increases it is imperative that the DC not only discuss the patient's current complaints, but also preventive strategies including fall prevention, nutritional counseling and physical activity. As the population continues to age there will be a greater need for the chiropractic profession to meet the needs of the older adult.

## Conclusions

While there is already substantial published research to assist the evidence-based DC in his/her care plan for the older adult, there is a need for well designed clinical trials and large observational studies to identify the most beneficial treatments, particularly for complementary and alternative interventions such as manual therapy including, but not limited to, spinal manipulative therapy and acupuncture.

## Competing interests

The authors declare that they have no competing interests.

## Authors' contributions

PD conceived of the study, designed the study, drafted the manuscript, and reviewed literature. CH participated in manuscript writing and literature review. DW participated in manuscript writing and literature review. BG participated in manuscript writing and literature review. KA participated in manuscript writing and literature review. LK participated in manuscript writing and literature review. All authors read and approved the final manuscript.
